# Clinical Characteristics Associated With Variability in Biopsy Grade Severity of Liver Fibrosis in Fontan Circulation

**DOI:** 10.1016/j.jacadv.2025.102421

**Published:** 2025-12-15

**Authors:** Taylor Hartzel Houlihan, Ari Gartenberg, Kathryn M. Dodds, Elizabeth B. Rand, Joseph Wu, Amy Roberts, Andrew C. Glatz, Michael O’Byrne, Pierre Russo, Benjamin Wilkins, Ann Marie Cahill, David J. Goldberg, Jack Rychik

**Affiliations:** a CS Mott Children’s Hospital, Department of Pediatrics, University of Michigan, Ann Arbor, Michigan, USA; b Division of Cardiology, Department of Pediatrics, Akron Children’s Hospital, Akron, Ohio, USA; c Division of Cardiology, Department of Pediatrics, Children’s Hospital of Philadelphia, Philadelphia, Pennsylvania, USA; d Division of Cardiology, Children's Hospital of Philadelphia and University of Pennsylvania School of Nursing, Philadelphia, Pennsylvania, USA; e Division of Hepatology, Gastroenterology and Nutrition, Department of Pediatrics, Children’s Hospital of Philadelphia, Philadelphia, Pennsylvania, USA; f Perelman School of Medicine, University of Pennsylvania, Philadelphia, Pennsylvania, USA; g Department of Biomedical and Health Informatics, Children’s Hospital of Philadelphia, Philadelphia, Pennsylvania, USA; h Division of Cardiology, Department of Pediatrics, Washington University St Louis Children’s Hospital, St. Louis, Missouri, USA; i Division of Anatomic Pathology, Department of Pathology and Laboratory Medicine, Children’s Hospital of Philadelphia, Philadelphia, Pennsylvania, USA; j Department of Pathology and Laboratory Medicine, Children’s Hospital of Philadelphia, Philadelphia, Pennsylvania, USA; k Division of Interventional Radiology, Department of Radiology, Children’s Hospital of Philadelphia, Philadelphia, Pennsylvania, USA

**Keywords:** Fontan associated liver disease, Fontan circulation, Fontan operation, single ventricle congenital heart disease

## Abstract

**Background:**

Liver fibrosis in Fontan circulation (FC) is universal with variable severity. We explore associations between patient-specific characteristics and fibrosis magnitude measured by liver biopsy.

**Objectives:**

The purpose of this study was to identify clinical and hemodynamic factors associated with fibrosis severity in FC, quantified by Sirius red staining of liver biopsy specimens, and to evaluate relationships with patient characteristics, Fontan hemodynamics, and noninvasive liver assessments.

**Methods:**

Subjects with FC underwent a standardized evaluation including cardiac catheterization, percutaneous liver biopsy, liver elastography, and blood laboratories. Sirius red % staining was used to quantitatively assess collagen deposition per liver tissue to measure fibrotic severity and associations with clinical characteristics explored.

**Results:**

Among 173 subjects, median age at Fontan operation (FO) was 2.2 years (range: 0.5-15), and median age at biopsy was 17.5 years (range: 4.7-39.4). Sixty-seven percent had dominant right ventricular morphology. Median systemic venous pressure at catheterization was 13 mm Hg (IQR: 11.0,15.0). Median Sirius red % staining was 21.6% (IQR: 17.3,30.2). Sirius red % staining was not associated with ventricular morphology, catheter hemodynamics, or liver elastography. Sirius red % staining correlated with aspartate aminotransferase/platelet ratio (*P* = 0.026). Years of FC exposure correlated positively with Sirius red % staining, hemoglobin, gamma glutamyl transferase, and liver elastography. Age at FO was inversely correlated with Sirius red % staining.

**Conclusions:**

Liver fibrosis in FC progresses with duration of exposure, independent of venous pressure, suggesting ongoing fibrogenesis even at acceptable hemodynamics. Younger age at FO is associated with greater fibrosis severity and may represent a modifiable factor influencing liver health.

While the Fontan operation (FO) allows for survival of individuals with single ventricle congenital heart disease, it comes at the cost of chronic systemic venous hypertension and limited cardiac output. This unique physiology creates a clinical state identified as a “Fontan circulatory syndrome,” with a host of end-organ consequences.^1^ Key among these is liver disease, which has emerged as a significant clinical problem. First reported in a pathology series in 2005,^2^ liver fibrosis is now appreciated to variable degrees in all individuals living with a Fontan circulation (FC) with a unique clinical signature. Liver function in the young is typically well preserved, with injury manifesting as modest elevations in blood liver enzymes and later thrombocytopenia.^3^^,^^4^ Liver imaging abnormalities are ubiquitous.^5^, ^6^, ^7^ Progressive fibrosis is suspected, and the incidence of cirrhosis increases with age.^8^ Severity of liver disease is associated with mortality^9^ and most dangerous is the risk of hepatocellular carcinoma, which can develop in young adults.^10^^,^^11^

A complete understanding of the mechanism of liver disease associated with FC remains elusive. Curiously, there is great variability in the magnitude of liver disease between individuals with a lack of knowledge concerning variables that contribute to this heterogeneity. Hepatic venous congestion likely plays an essential role, yet the degree of venous congestion alone does not fully explain the variability. Part of the challenge is characterizing the magnitude of liver disease in an accurate and consistent manner, as controversy exists concerning the optimal tests. Confounding this challenge is the unique state of liver stress which includes hepatic vascular congestion and tissue fibrosis.^12^ Although imperfect, direct inspection of liver biopsy specimens is one means of gauging the magnitude of liver fibrosis.^13^

Investigating the relationship between measures of liver fibrosis and clinical characteristics can provide insights into the origins of liver fibrosis variability seen in FC. In a dedicated Fontan follow-up clinic at our center—the Fontan rehabilitation, wellness and resilience development [FORWARD] program,^14^ we offered a consistent, standardized approach of detailed liver characterization. Incorporation of American Heart Associations statement recommendations^1^ for serial clinical characterization into a surveillance strategy with standardized liver evaluation including percutaneous liver biopsy with quantitative means of measuring fibrosis has given us the opportunity to explore clinical variables associated with the magnitude of liver fibrosis in FC. The purpose of this study is to explore associations and identify patient-specific characteristics that may influence the magnitude of liver fibrosis measured by liver biopsy in individuals with FC and thus gain insights into mechanistic origins and nature of the disease.

## Methods

This study consists of a retrospective review of single ventricle patients who underwent comprehensive evaluation through the FORWARD program at Children’s Hospital of Philadelphia between 2008 and 2022. Following an internal consensus conference, our center decided to offer comprehensive evaluation including cardiac catheterization and liver biopsy to all individuals at 10 years out from FO as part of routine care and at discretion of individual primary cardiologists.^15^ Evaluation included echocardiography, cardiopulmonary exercise testing, cardiac catheterization, liver biopsy, liver ultrasound shear-wave elastography,^16^ and laboratory work (complete blood count, liver function tests, and B-natriuretic peptide). All data were collected as part of standard clinical surveillance in the FORWARD program, including liver biopsy. All subjects were clinically well outpatients, referred to undergo elective FORWARD program evaluation. Permission to review the data and analyze for investigational purposes was granted under Children’s Hospital of Philadelphia Institutional Review Board (IRB 12-00979 and IRB 20-017749).

Liver biopsies were collected percutaneously at the time of cardiac catheterization or within 3 months of each other when rarely performed separately.^17^ Tissue samples were stained with Sirius red stain. Quantification of liver fibrosis was performed by pathologists blinded to clinical data by calculating the percentage surface area stained with Sirius red, a fibrosis collagen-specific marker, as described previously.^13^ Sirius red staining allows for a quantitative means to assess and grade *total* fibrotic load and is of particular benefit in the FC state where a mixed picture of centrilobular and perisinusoidal fibrosis is typically present to variable degrees.

All patients with liver biopsies and cardiac catheterizations as well as complete Fontan FORWARD clinic visits were included in the analysis. The dates of Fontan surgical palliation, cardiac catheterization hemodynamic values, liver elastography results, percentage of Sirius red staining, and laboratory values including platelets, hemoglobin, alanine transaminase, aspartate transaminase, gamma glutamyl transferase (GGT), international normalized ratio, and B-natriuretic peptide obtained around the time of liver biopsy were included in the analysis.

### Statistical analysis

A statistical analysis plan was developed before assessing the data. No statistical power analysis was conducted, and the sample size was a convenience sample based on the patient volume available. Descriptive statistics were used to present demographic and clinical characteristics across the quartiles of FC duration, categorical variables were described using count and percentage, and the numeric variables were described using median and IQR (the normality assessed using the Shapiro-Wilk test and histogram indicating skewed distributions for all numeric variables). The missing data were also reported.

Correlation between the primary outcome, the quantitative percentage of positive Sirius red reflecting magnitude of liver fibrosis, and numeric clinical measures including hemodynamic parameters and laboratory measures was assessed using Spearman correlation coefficient. The outliers and monotonicity were examined using scatterplots. With detected skewed distributions and possible outliers, Spearman rank correlation coefficient was therefore used. Correlation was also assessed between duration of FC and numeric variables. A multivariable generalized linear regression model was built to assess the association of the outcome variable with Fontan pressure and duration of FC. Natural-log transformation of the primary outcome variable was performed given its skewed distribution. The model was fitted with a normal (Gaussian) distribution and identity link function and adjusted for age at Fontan, single ventricle type, heterotaxy, hypoplastic left heart syndrome (HLHS), pacemaker, and rhythm. The selection of the adjusted covariates was based on the clinical conceptual model. The multicollinearity was assessed using variance inflation factor and no obvious collinearity was detected. Exponentiated regression coefficients along with the 95% CIs were reported. The distribution of key continuous variables was shown using histograms. A scatterplot was drawn: the log-transformed percentage of positive Sirius red was plotted against the FC duration with a linear regression line fitted to indicate the corresponding trend. The Fontan pressure-time parameter (Fontan pressure x years of FC) was also assessed as an interaction term incorporated. A scatterplots panel stratified by the quartiles of Fontan pressure was created presenting whether the trends between Sirius red and Fontan duration varied across levels of Fontan pressure. Both a LOESS (locally estimated scatterplot smoothing regression algorithm) curve and a linear regression line were added. *P* value <0.050 was considered of significance. Statistical analyses were performed using R version 4.4.1 (R Foundation for Statistical Computing).

## Results

### Subject population characteristics

A total of 173 subjects were included in the study. Table 1 describes the demographics for the cohort and characteristics of groups divided into quartiles by years of living with a FC. The median age at the time of liver biopsy was 17.5 years with a range of age 4.7 to 39.4 years. The majority were male (61%) and White (80%). The median age at FO was 2.2 years with a range of 0.5 to 15 years. More patients had a dominant single right ventricle morphology (67%) as opposed to left ventricle (33%) with 46% of patients diagnosed with HLHS. A small percentage of patients had pacemakers (9.3%). The median Sirius red liver stain percentage was 21.6% (IQR: 17.3, 30.2). The median highest central venous pressure (inferior vena cava, Fontan pathway, or branch pulmonary artery) measured during catheterization was 13 mm Hg (IQR: 11.0, 15.0).

**Table 1:** Characteristics of the Patient Cohort Overall and by Years With FC

Characteristic	Overall N = 173	Four Quartiles of Years With FC			
		1st Q: 3.1 to 11.6 Years N = 43	2nd Q: 11.7 to 14.6 Years N = 42	3rd Q: 14.7 to 18.1 Years N = 44	4th Q: 18.2 to 30 Years N = 44
Sex					
Female	67 (39%)	21 (49%)	12 (29%)	18 (41%)	16 (36%)
Male	106 (61%)	22 (51%)	30 (71%)	26 (59%)	28 (64%)
Race and ethnicity					
White	138 (80%)	34 (79%)	35 (83%)	35 (80%)	34 (77%)
African American	16 (9.2%)	5 (12%)	2 (4.8%)	4 (9.1%)	5 (11%)
Hispanic	9 (5.2%)	1 (2.3%)	3 (7.1%)	2 (4.5%)	3 (6.8%)
Other	10 (5.8%)	3 (7.0%)	2 (4.8%)	3 (6.8%)	2 (4.5%)
Age at Fontan (y)	2.2 (1.7, 3.3)	2.9 (2.0, 3.5)	2.2 (1.8, 3.0)	1.9 (1.6, 2.5)	2.0 (1.5, 3.8)
Single ventricle type					
LV	56 (33%)	9 (21%)	11 (26%)	15 (34%)	21 (48%)
RV	116 (67%)	33 (79%)	31 (74%)	29 (66%)	23 (52%)
Unknown	1				
Hypoplastic left heart syndrome (HLHS)					
Non-HLHS	94 (54%)	15 (35%)	18 (43%)	29 (66%)	32 (73%)
HLHS	79 (46%)	28 (65%)	24 (57%)	15 (34%)	12 (27%)
Heterotaxy syndrome	17 (9.8%)	5 (12%)	2 (4.8%)	6 (14%)	4 (9.1%)
Pacemaker					
No pacemaker	156 (91%)	42 (98%)	37 (90%)	39 (89%)	38 (86%)
Pacemaker	16 (9.3%)	1 (2.3%)	4 (9.8%)	5 (11%)	6 (14%)
Unknown	1				
Rhythm at the time of cardiac catheterization					
Nonsinus	30 (17%)	3 (7.0%)	10 (24%)	7 (16%)	10 (23%)
Sinus	142 (83%)	40 (93%)	31 (76%)	37 (84%)	34 (77%)
Unknown	1				
Age at the time of cardiac catheterization (y)	17.2 (14.2, 20.8)	13.2 (11.5, 13.9)	15.4 (14.8, 16.0)	18.8 (17.6, 19.3)	23.5 (21.7, 30.2)
Highest CVP in FC (mm Hg)	13.0 (11.0, 15.0)	13.0 (12.0, 15.0)	13.0 (12.0, 15.0)	12.0 (10.0, 13.0)	13.0 (11.5, 16.0)
Age at liver biopsy (years)	17.5 (14.2, 20.8)	13.2 (11.4, 13.9)	15.6 (14.8, 16.0)	18.9 (17.7, 19.3)	23.4 (21.9, 30.2)
Positive Sirius red, %	21.6 (17.3, 30.2)	20.0 (13.5, 29.3)	21.3 (19.1, 26.2)	20.5 (16.8, 30.3)	25.1 (18.3, 34.9)
n (%); median (IQR)					

CVP = central venous pressure; FC = Fontan circulation; LV = left ventricle; RV = right ventricle.

To determine differences in the study population based on years of exposure to FC, we divided the cohort by quartiles of years living with a FC (quartile 1 = 3.1-11.6 years, quartile 2 = 11.7-14.6 years, quartile 3 = 14.7-18 years, and quartile 4 = 18.2-30 years). Patient demographics such as sex and race/ethnicity did not differ; however there was a greater percentage of right ventricle dominant single ventricle type and HLHS patients in the lower quartiles 1 and 2 (majority of subjects), than in the upper quartiles 3 and 4. Age at FO was slightly older in the lower quartiles 1 and 2 than in the upper quartiles 3 and 4. Fontan pathway pressures did not differ by quartile. Nonsinus rhythm at the time of catheterization was less frequent in quartile 1 compared to quartiles 2, 3, and 4, and frequency of pacemakers increased with each quartile.

### Catheterization data

Cardiac catheterization data were analyzed to explore any significant correlations between 1) percentage of Sirius red staining and FC hemodynamics and 2) number of years of exposure to FC and FC hemodynamics (Table 2). There were no significant correlations found between Sirius red staining and catheter hemodynamics. Sirius red staining only correlated significantly with years of exposure to FC (correlation = 0.162; *P* = 0.033). However, increased years of exposure to FC correlated significantly with increased aortic mean pressure (correlation = 0.231; *P* = 0.005), decreased superior vena cava oxygen saturation (correlation = −0.179; *P* = 0.020), decreased pulmonary blood flow index (correlation = −0.211; *P* = 0.007), and decreased systemic cardiac index (correlation = −0.347; *P* < 0.001). Notably, years of exposure to FC did not correlate with any Fontan pathway pressures.

**Table 2:** Correlations of Hemodynamics to % Positive Sirius Red and Years of FC

Measures	Median (IQR)	n	Positive Sirius Red, %		Years of FC	
			Spearman correlation	*P* Value	Spearman correlation	*P* Value
Positive Sirius red, %	21.6 (17.3, 30.2)	173	1.000		0.162	***0.033***
Years of FC	14.7 (11.7, 18.2)	173	0.162	***0.033***	1.000	
Highest CVP in FC (mm Hg)	13.0 (11.0, 15.0)	170	0.022	0.776	−0.027	0.725
IVC pressure (mm Hg)	13.0 (11.0, 15.0)	163	0.012	0.876	−0.056	0.474
SVC pressure (mm Hg)	13.0 (11.0, 15.0)	171	0.022	0.778	−0.048	0.529
LPA pressure (mm Hg)	12.0 (10.0, 14.0)	172	0.026	0.730	−0.044	0.567
RPA pressure (mm Hg)	12.0 (10.0, 14.0)	167	0.008	0.922	−0.101	0.195
Aortic mean pressure (mm Hg)	69.0 (63.0, 78.8)	146	0.029	0.724	0.231	***0.005***
SV end-diastolic pressure (mm Hg)	8.0 (7.0, 10.0)	130	0.081	0.358	0.005	0.954
Mean PCWP pressure (mm Hg)	9.0 (7.0, 10.0)	173	0.049	0.525	−0.045	0.560
SVC saturation, %	70.0 (65.0, 73.0)	167	−0.036	0.644	−0.179	***0.020***
Aorta saturation, %	92.0 (89.0, 94.0)	163	0.021	0.792	−0.071	0.366
RPA saturation, %	71.0 (68.0, 74.0)	148	0.001	0.991	−0.045	0.586
LPA saturation, %	72.0 (68.0, 75.0)	168	0.019	0.811	0.031	0.690
Pulmonary (Qp) index	2.6 (2.4, 3.2)	163	−0.028	0.720	−0.211	***0.007***
Systemic cardiac (Qs) index	3.0 (2.6, 3.6)	172	−0.026	0.735	−0.347	***<0.001***

IVC = inferior vena cava; LPA = left pulmonary artery; PCWP = pulmonary capillary wedge pressure; RPA = right pulmonary artery; SV = single ventricle; SVC = superior vena cava; other abbreviation as in Table 1.

### Laboratory and elastography data

Liver injury, function blood labs, and ultrasound-derived shear-wave liver elastography values were analyzed to explore for any significant correlations between percentage of Sirius red staining and 1) clinical laboratory measures of liver disease and 2) number of years of exposure to FC (Table 3). Of all the blood laboratories, Sirius red staining only correlated significantly with aspartate aminotransferase (AST)/platelet count ratio (correlation = 0.184; *P* = 0.026). Years of exposure to FC correlated significantly with increased hemoglobin levels (correlation = 0.276; *P* < 0.001), GGT (correlation = 0.279; *P* = 0.001), and GGT/platelet count ratio (correlation = 0.311; *P* = 0.001). Sirius red % staining did not correlate with ultrasound liver elastography. Years of Fontan exposure correlated significantly with ultrasound liver elastography (correlation = 0.274; *P* = 0.034).

**Table 3:** Correlations of Lab Measures to Positive Sirius Red and Years of FC

Measures	Median (IQR)	n	Positive Sirius Red, %		Years of FC	
			Spearman correlation	*P* Value	Spearman correlation	*P* Value
Platelet	183.5 (150.0, 229.2)	148	−0.097	0.241	−0.152	0.066
Hemoglobin	15.7 (14.6, 16.7)	164	−0.023	0.771	0.276	***<0.001***
ALT	33.0 (24.0, 45.0)	169	−0.013	0.863	−0.052	0.502
AST	41.0 (33.0, 49.0)	171	0.135	0.078	−0.039	0.610
GGT	62.5 (44.0, 100.8)	134	0.155	0.075	0.279	***0.001***
INR	1.2 (1.2, 1.3)	148	0.099	0.232	0.081	0.326
B-natriuretic peptide	25.6 (16.1, 45.0)	106	0.156	0.110	−0.001	0.992
AST/Platelet count x 100	21.5 (15.7, 29.9)	147	0.184	***0.026***	0.069	0.410
GGT/platelet count x 100	30.4 (21.1, 56.1)	116	0.153	0.101	0.311	***0.001***
Liver elastography (m/sec) median	2.1 (1.7, 2.6)	60	0.005	0.971	0.274	***0.034***

ALT = alanine aminotransferase; AST = aspartate aminotransferase; GGT = gamma glutamyl transferase; INR = international normalized ratio; other abbreviation as in Table 1.

Patients with heterotaxy were excluded for measures involving platelets.

### Multivariate analysis

Multivariate analysis was performed to evaluate associations between percentage of Sirius red staining and clinical variables suspected to impact liver fibrosis (Table 4). Two models were tested: Model I with years of Fontan exposure and highest Fontan pressure as separate variables, and Model II with the multiple of Fontan pressure x years of exposure to FC as a variable. After controlling for multiple covariates, a statistically significant correlation between years of exposure to FC and Sirius red staining was found (Exp = 1.017; *P* = 0.014). Thus, for each year of FC exposure, the percentage of positive Sirius red significantly increased by an average of 1.7% (95% CI: 0.04%-3.1%). The only other significant variable found in both models was age at FO. This was *negatively* associated with percentage of Sirius red staining suggesting that for each increased year of age at Fontan surgery, the percentage of Sirius red staining decreased by 3.8% (95% CI from 0.1% to 7.4%; *P* = 0.049).

**Table 4:** Multivariable Associations Between Percentage of Positive Sirius Red and Risk Factors

Features	Model I			Model II		
	Exp(B)	95% CI	*P* Value	Exp(B)	95% CI	*P* Value
Years since FC	***1.017***	(***1.004***-***1.031***)	***0.014***			
Fontan pressure: highest CVP in FC	1.017	(0.995-1.038)	0.130			
Fontan pressure x years of FC				***1.001***	(***1.000***-***1.002***)	***0.001***
Age at Fontan	***0.962***	(***0.926***-***0.999***)	***0.049***	***0.956***	(***0.920***-***0.994***)	***0.026***
Single ventricle type: RV vs LV	0.876	(0.711-1.080)	0.216	0.876	(0.713-1.077)	0.210
Heterotaxy: yes vs no	1.161	(0.885-1.523)	0.284	1.163	(0.889-1.522)	0.272
Hypoplastic left heart syndrome: HLHS vs non-HLHS	1.152	(0.937-1.416)	0.181	1.149	(0.939-1.405)	0.181
Pacemaker vs no pacemaker	0.936	(0.682-1.284)	0.682	0.936	(0.684-1.282)	0.682
Rhythm: sinus vs nonsinus	1.124	(0.885-1.427)	0.339	1.130	(0.892-1.432)	0.312

Abbreviation as in Table 1.

**Figure 1:**
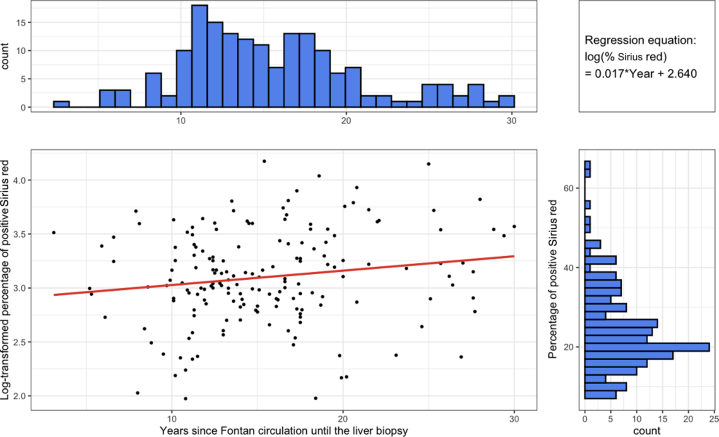
**Association Between Years of Fontan Circulation and Sirius Red Staining** Log-transformed scatterplot showing a positive relationship between years of FC and Sirius red staining. Boxplots on the X and Y axes display distributions of years of FC exposure and percentage of positive Sirius red stain, respectively. A mild upward trend is observed. FC = Fontan circulation.

**Figure 2:**
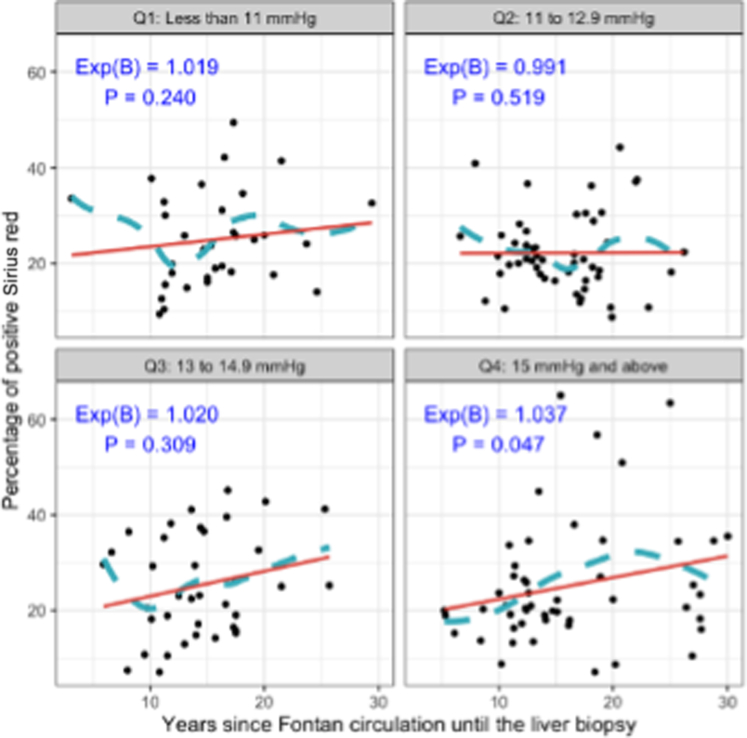
**Impact of Fontan Systemic Venous Pressure on Sirius Red Staining** Stratified analysis by Fontan systemic venous pressure quartiles shows a significant positive association between percentage Sirius red staining and years of FC exposure only in the highest-pressure group (≥15 mm Hg). In this group, Sirius red staining percentage increases by 3.7% per year of FC exposure. Abbreviation as in Figure 1.

Within the study population, the percentage of Sirius red staining had a skewed distribution. Logarithmic transformation was performed to generate a linear model which demonstrates the positive relationship between years of FC and Sirius red staining (Figure 1). There was a statically significant positive correlation between the Fontan pressure multiplied by years of FC parameter and Sirius red staining (Exp = 1.001; *P* = 0.001) although Fontan central venous pressure alone did not correlate significantly with staining percentage. When Fontan central venous pressures were broken into quartiles (Figure 2), there was a significant positive association between the years of exposure to FC and the percentage of positive Sirius red staining in the fourth quartile of highest Fontan pressures ≥15 mm Hg (Exp = 1.037; *P* = 0.047), but not in the lower quartiles of lower Fontan pressures <15 mm Hg. For those with Fontan pressures ≥15 mm Hg, percentage of Sirius red staining increased by around 3.7% with each additional year of exposure to FC.

## Discussion

All individuals with FC experience liver disease; however, the severity and clinical manifestations within the population vary widely. The etiology of liver disease is likely multifactorial including alterations in blood flow prenatally due to congenital heart malformations, insults from cardiac surgeries, chronic hypoxia, and long-standing stressors of Fontan physiology. These factors cumulatively contribute to the potential for liver injury and promotion of fibrogenesis. To date, clinical evaluation strategies for liver disease in the FC vary across centers, and factors that contribute to the disease remain elusive.

In this study, we report on a large single-center series of individuals with FC who underwent systematic evaluation including cardiac catheterization and liver biopsy. Despite the invasive nature of this strategy, we felt it important to offer this intensive surveillance through an institutional group consensus.^15^ A proactive approach, instead of a reactive one, might offer a better chance to stave off and prevent complications as individuals enter their formative adolescent and early adult years. We established a program of standardized, invasive evaluation at a life trajectory milepost of 10 years from FO to provide accurate characterization and a “snapshot” of status, allowing for individualized care and prognosis. Furthermore, we undertook a standardized quantitative approach to the assessment of liver tissue fibrosis by the Sirius red percent staining method. This technique measures total burden of liver fibrosis in a continuous variable manner and may be more useful than other means of categorical grading to analyze against clinical variables and help tease out associations. After enacting this programmatic, standardized approach for several years, we can now explore clinical associations with degree of fibrosis in this large cohort, and thus identify factors influencing variability, gain insights into mechanistic origins, and create opportunities to potentially modify the disease.

Does Fontan central venous pressure influence severity of liver fibrosis? Intuitively, one might expect this to be the case, however for the overall cohort there was no relationship found. Time of exposure to the Fontan physiology, however, is a significant factor in determining magnitude of liver fibrosis. While alluded to in our prior work,^18^ we now further demonstrate this phenomenon in a much larger cohort with additional opportunity for identifying clinical variables associated. Our findings confirm that despite the central venous pressure, exposure to any degree of Fontan circulatory physiology in a sustained manner drives fibrosis. Of note, when looking at the study population broken down by quartiles of time exposure to a FC, there were no significant differences in Fontan central venous pressures across groups. However, when we analyzed the time exposure in the subgroup with a Fontan circulatory pressure ≥15 mm Hg, we found a more substantial impact on the rate of fibrotic change, with an increase in Sirius red staining of 3.7% per year of exposure. This suggests that while time of exposure is a key contributing element to fibrosis at all Fontan pressures, it is accentuated when pressures are elevated beyond 15 mm Hg. This was confirmed by the strong association between the composite variable of the product of years of Fontan exposure and Fontan central venous pressure vs liver fibrosis in multivariate Model II.

While there were no hemodynamic associations for the overall cohort with liver fibrosis, there were some hemodynamic associations with years of FC exposure. Aortic mean pressure was higher, and superior vena cava saturation, pulmonary and systemic blood flows were all lower, with increasing time of exposure (Central Illustration). These findings are consistent with the general trend toward altered hemodynamics and increased systemic vascular resistance over time in those with FC.^19^, ^20^, ^21^

**Central Illustration:**
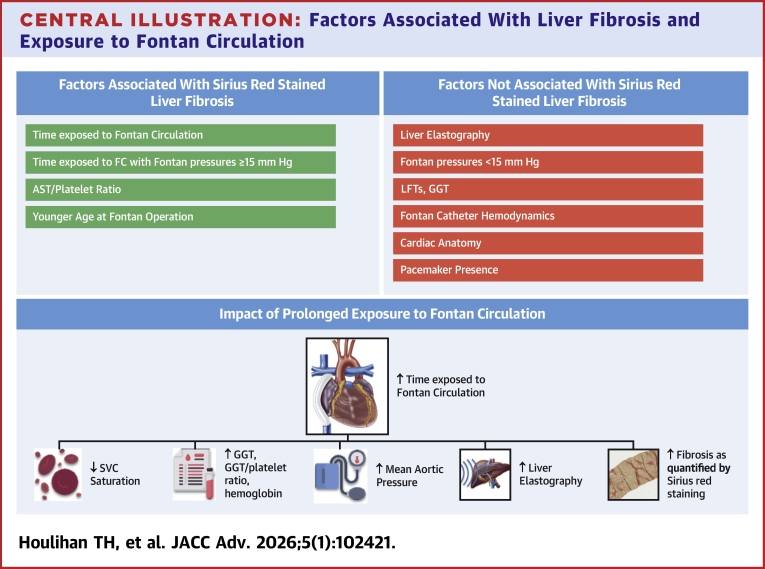
**Factors Associated With Liver Fibrosis and Exposure to Fontan Circulation** Liver fibrosis as quantified by Sirius red staining was significantly associated with select cardiac variables (top left), but not other hemodynamic or laboratory markers (top right). Increased exposure to FC was significantly associated with fibrotic, laboratory, and hemodynamic changes (bottom). ALT = alanine aminotransferase; AST = aspartate aminotransferase; GGT = gamma glutamyl transferase; LFTs = liver function tests; SVC = superior vena cava; other abbreviation as in Figure 1.

Blood laboratory assessments are now commonly included in the serial surveillance of individuals with FC. Liver function blood laboratories are often moderately elevated, and platelet counts moderately diminished.^4^^,^^22^ Of the clinical laboratory variables collected in our study, only the AST/platelet count ratio was significantly associated with magnitude of biopsy-measured liver fibrosis. The “AST-to-platelet ratio index” or APRI has been reported as a useful noninvasive measure of fibrosis in other liver conditions such as chronic hepatitis C, as well as in a pediatric population of biliary atresia.^23^^,^^24^ We suggest the APRI should be explored further as a candidate biomarker for liver fibrosis in the FC, as other investigators have similarly identified this as a potentially valuable index.^25^ Of the blood labs, hemoglobin values also increased with Fontan time exposure, likely related to changes in systemic oxygen saturation over time.

Interestingly, GGT was not associated with magnitude of liver fibrosis, but was highly associated with time exposure to FC. Some investigators using platelet count itself as a measure of liver fibrosis have suggested a relationship between GGT and liver fibrosis in the FC, but this study, as are others, is faced with the limitation of comparing indirect biomarkers to each other, not with liver biopsy data.^26^ GGT release is now appreciated to reflect a variety of regulatory processes, including the response to chronic heart failure via upregulation of cellular oxidative stress.^27^, ^28^, ^29^ The Fontan circulatory syndrome certainly falls into this category. The time-based elevations in GGT we found may be less specific to liver fibrosis than to the biological response to circulatory insufficiency present in the FC. We propose that GGT values are not solely specific to liver disease but rather released due to cardiovascular stressors, as it tracks closely with time of Fontan exposure and *not* magnitude of liver fibrosis. GGT is a fascinating ubiquitous biomarker and is worthy of further exploration as it may be informative more broadly of the biology of the FC and perhaps of future prognosis.

Noteworthy are some of the interesting absences of associations in our study. Anatomical features such as dominant single ventricle morphology type or heterotaxy syndrome were not associated with liver fibrosis. HLHS, with its diagnosis reflecting a more rigorous early operative course including newborn Norwood operation, was not associated with degree of liver fibrosis. Absence of sinus rhythm is speculated to be a risk factor in the FC, as loss of atrioventricular synchrony may diminish ventricular filling and impede hepatic venous drainage causing worsening congestion.^30^ Interestingly, nonsinus rhythm was present in 17% of our study cohort and was not associated with degree of liver fibrosis in our multivariate analysis. From the diagnostic perspective, ultrasound elastography did not correlate to the degree of liver fibrosis. Liver stiffness measures are a composite of hepatic vascular congestion and liver tissue fibrosis, both of which are present in FC. Standard elastography techniques cannot distinguish between these,^12^ however more sophisticated tools such as three-dimensional magnetic resonance imaging elastography or newer magnetic resonance imaging sequences specific for collagen burden may provide future promise.^31^, ^32^, ^33^ Of note, liver elastography measures were associated with years of Fontan exposure, further suggesting the measure may be confounded by other factors besides liver fibrosis alone. Since liver elastography is a factor of both liver fibrosis and vascular congestion, it could potentially be a valuable measure reflecting a composite, which incorporates both of these critical features into an index that characterizes the overall Fontan circulatory state.

Finally, one of the most impactful and surprising findings in our study is the relationship between time of performance of the FO and liver fibrosis. In both multivariate models, we found that age at FO was significantly inversely related to magnitude of liver fibrosis. Thus, when controlling for exposure time to FC, the younger the individual at the time of Fontan surgery, the greater the degree of liver fibrosis later in life. Given the narrow window of surgical performance of the FO early in life, this is a remarkable finding. It is well established that the liver develops and matures rapidly during the first 2 years of life.^34^, ^35^, ^36^ It is plausible to suspect that an earlier imposition of the rigors of FC during a vulnerable and fragile period of liver development may have untoward negative long-term consequences, manifesting as a greater degree of liver fibrosis later in life. Maturation of the gut microbiome may also contribute to a more favorable time for imposition of FC from a liver health perspective.^37^ This finding supports a provocative argument for moving the typical electively timed completion of the FO^38^ to a later point beyond 2 years of age, thus exposing a more mature and perhaps more resilient liver to the long-standing effects of the FC.

### Study limitations

There are several limitations to our study. This is a cross-sectional analysis of a large cohort, so while exposure time to FC for the group overall is significant, only serial longitudinal data can best answer the question of the impact of exposure time to FC for any one individual. The sample size of the study is relatively small with limited power of detecting the significant differences. This limitation has likely affected the analysis with nonsignificant correlations identified in the study. The issue of small sample size may also introduce random errors to the analysis resulting in larger CIs. Nevertheless, this study is one of the largest series of biopsy demonstrated liver fibrosis with concurrent systematic clinical laboratory characterization reported to date. Given these limitations, our study discovers “signals” of specific clinical variables among a myriad of clinical “noise” that now allows for more focused exploration of factors influencing magnitude of liver fibrosis. Catheterization hemodynamics were obtained in a supine, sedated state and likely do not reflect the upright, ambulatory awake state in which most subjects exist. We explore the Fontan pressure—years of circulation multiple as a variable, however this assumes stable Fontan pressure over this period of time, which is unlikely. A means for noninvasively measuring hemodynamics variability and vascular congestion in real-life would be valuable in the FC but evades us at this time. Although liver biopsy is arguably the best measure we have for characterizing fibrosis, it is an imperfect tool. Liver fibrosis in FC can be heterogeneous, resulting in sampling error. In order to overcome regional tissue bias which arises from a transvenous approach where perivascular tissue may be preferentially sampled, we performed percutaneous liver biopsies, using a two-pass ultrasound-guided technique. Nevertheless, we accept as a fair critique that regional tissue biopsy may not represent whole organ status. Also, while % Sirius red staining may not be a common method of liver pathology characterization in other conditions, we reported its utility specific to the FC. The method allows for semiautomated optical photometry for objectively quantifying the amount of fibrosis in a field of view and provides for a continuous variable value a opposed to categorical grades seen in other scoring systems and is therefore a reasonable endpoint to compare against a host of clinical variables. Finally, it is important to appreciate the select nature of the cohort studied and the potential for bias. Subjects were included only after referral to the FORWARD program for elective surveillance evaluation and likely represent the healthiest among the population. All were outpatients, and none were on the transplant list. Our findings therefore reflect “best case” scenarios of routine FC hemodynamics and their impact on liver fibrosis. Additional conditions, such as severe ventricular dysfunction or atrioventricular valve regurgitation, or presence of clinically active lymphatic insufficiency syndromes like plastic bronchitis or protein-losing enteropathy may add additional risks on top of the baseline impact we describe.

## Conclusions

Magnitude of Fontan central venous pressure *is not*, however the extent of time exposed to the FC *is* associated with magnitude of liver fibrosis, suggesting that even at conventionally acceptable levels of Fontan central venous pressure, the process of fibrosis is ongoing. Anatomical subtypes of single ventricle or absence of sinus rhythm are not associated with degrees of liver fibrosis. The APRI may be a promising noninvasive tool for gauging liver fibrosis, while GGT is not associated with liver fibrosis but increases with age and may reflect other processes. Ultrasound liver elastography is poorly specific for magnitude of liver fibrosis, however liver stiffness may reflect the combined effect of fibrosis and congestion which may be valuable to monitor serially. Younger age at FO increases the risk for magnitude of liver fibrosis and is a modifiable variable in the overall strategy for improving liver health in FC. Exploring the relationships between these clinical characteristics and biological aspects unique to Fontan-associated liver disease^39^, ^40^, ^41^ will yield new insights that translate into better understandings and targeted therapies to treat this condition.

## Funding support and author disclosures

Dr Rychik’s efforts as well as funding for this work is supported by the Robert & Dolores Harrington endowed Chair in Cardiology at Children’s Hospital of Philadelphia. The authors have reported that they have no relationships relevant to the contents of this paper to disclose.
